# CircLATS2 Regulates miR-520a-3p/E2F7/p-VEGFR2 Signaling Pathway to Promote Hepatocellular Carcinoma Progression and Angiogenesis

**DOI:** 10.1155/2022/3744560

**Published:** 2022-04-11

**Authors:** Yefeng Wu, Jianmao Yuan, Zhengbin Tu, Huahua Chen

**Affiliations:** ^1^Hepatobiliary Surgery, Suzhou Ninth People's Hospital, Suzhou, Jiangsu 215200, China; ^2^Department of Stomatology, Suzhou Ninth People's Hospital, Suzhou, Jiangsu 215200, China

## Abstract

**Objective:**

To investigate the effect of circLATS2 on the progression and angiogenesis of hepatocellular carcinoma and its molecular mechanism.

**Methods:**

The expression of circLATS2 in hepatocellular carcinoma was detected by qRT-PCR. The StarBase database was used to predict the potential miRNA, and the combination of the above was cytological verified by luciferase reporter gene assay and RNA pull down. The potential target genes of miRNA were predicted by TargetScan, verified by the above experiments, and the influence of circLATS2 on its expression was determined. The biological function of circLATS2 was investigated by in vitro and in vivo experiments. The effects of miRNA and target genes on the malignant behavior of HCC cells were determined by the reverse experiment.

**Results:**

circLATS2 was highly expressed in HCC and was positively correlated with tumor size and tumor stage. miR-520a-3p was sponged by circLATS2 and was low expressed in HCC tissues. As the target gene of miR-520a-3p, the expression level of E2F7 is affected by circLATS2. In vitro experiments showed that circLATS2 knockdown inhibited the proliferation, clone formation, migration, and invasion ability of hepatocellular carcinoma cells. In vivo knockdown of circLATS2 inhibits the proliferation of HCC cells, while overexpression of circLATS2 promotes the proliferation of HCC cells. Overexpression of miR-520a-3p and E2F7 knockdown reversed the role of circLATS2 in promoting malignant behavior of HCC cells and affected phosphorylation of VEGFR2.

**Conclusion:**

CircLATS2 promotes the progression of HCC by regulating miR-520a-3p/E2F7/P-VEGFR2 signaling pathway.

## 1. Introduction

Hepatocellular carcinoma (HCC) is the most common type of cancer worldwide and the fourth leading cause of cancer-related deaths [1]. More than 90% of HCC cases are hepatocellular carcinoma, and chemotherapy and immunotherapy are the best choices. Only 5%-15% of these patients diagnosed in the early stages are able to be treated surgically, whereas in the later stages only transchemoembolization (TACE) is performed, or sorafenib is taken oral [2, 3]. However, less than one-third of patients can benefit from treatment, so finding better therapeutic targets is crucial for the prognosis of patients with advanced HCC. Vascular endothelial factor (VEGF) is an important angiogenic cytokine, and VEGF signaling pathway plays an important role in tumor angiogenesis and tumor progression of HCC [4, 5]. Current systemic therapies, such as sorafenib and lumvaritinib, target VEGF and its receptor angiogenesis as first-line therapies [6].

Circular RNAs (CircRNA) are a class of single-stranded RNA molecules, which can lead to novel backsplicing or skipping events from precursor mRNAs [7]. Many studies have shown that circRNA plays a key role in the regulation of angiogenesis and is abnormally expressed in a variety of tumors. Xu et al. found that circRNA-SORE was upregated in sorafenib drug-resistant hepatocellular carcinoma cells, which blocked PRP19-mediated YBX1 degradation by binding to cytoplasmic oncogenic protein YBX1. Moreover, silencing circRNA-SORE can significantly overcome sorafenib resistance in animal models [8].

In lung cancer, Chen et al. found that circRNA100146 promoted the proliferation, invasion, and angiogenesis of cancer cells by binding miR-361-3p and miR-615-5p [9]. Has-circ-0007534 was shown to be significantly upregulation in breast cancer cells, promoting the proliferation and angiogenesis of breast cancer cells [10]. Relevant studies have also been carried out in HCC. Huang et al. found that has_circRNA_104348 was significantly upregulated in hepatocellular carcinoma tissues and cells and was associated with poor prognosis of patients. This effect promotes the proliferation, migration, invasion, and apoptosis of HCC cells by targeting miR-87-3p to regulate Wnt/*β*-catenin signaling pathway [11]. Therefore, circRNA plays an important role in the angiogenesis of HCC, and it is of practical significance to gradually clarify its regulatory mechanism for the understanding of HCC. Studies have shown that in breast cancer cells, circLATS2 promotes cell proliferation through miR-4686/WNT5A axis. However, the role of circLATS2 in hepatoma cells has not been reported. In this study, the function of circLATS2 in HCC was studied, and the prediction of Starbase and TargetScan websites revealed that miR-520a-3p may be the target gene of circLATS2. E2F7 may be the target gene of miR-520a-3p.

E2F3 belongs to the E2F transcription factor family; E2F1 and E2F3 are involved in maintenance and self-renewal of cancer stem cells [12]. Dysregulation of E2F transcriptional activator is found in a variety of cancers, including bladder, breast, and ovarian cancers [13–16]. E2F3 is abnormally upregulated in hepatocellular carcinoma and promotes the proliferation and invasion of tumor cells. Vascular endothelial growth factor (VEFGR) exerts its activity by binding to VEGFR. VEGFR2 regulates endothelial cell proliferation, migration, differentiation, and vascular formation by mediating the primary receptor of VEGF angiogenic activity [17]. HCC is considered to be one of the most vascular solid tumors. VEFGR2, as a target of angiogenesis, influences the progression of HCC [18].

In this study, the molecular mechanism of the influence of circLATS2 on HCC progression and angiogenesis, as well as the effect of circLATS2 on VEFGR2-related signaling pathways, provides potential therapeutic targets for the treatment of HCC.

## 2. Method

### 2.1. Cell Culture

Human hepatocellular carcinoma cell lines PLC-PRF-5 and HepG2 were purchased from the American Type Culture Collection. Cells were cultured in DMEM medium containing 10% fetal bovine serum (including 100 U/mL and 100 *μ*g/mL streptomycin (Sigma, St. Louis, MO, USA)) at 37°C and 5% CO_2_. PLC-PRF-5 is human hepatocellular carcinoma Alexander cell line, which secretes hepatitis B virus surface antigen (HBsAg). HepG2 cells were derived from liver cancer tissue of a 15-year-old white man. The cell secretes various plasma proteins. It also expresses alpha-fetoprotein and has the activities of 3-hydroxy-3-formyl-coA reductase and hepatic triglyceride lipase.

### 2.2. Cell Transfection

Genechem (Shanghai, China) synthesized short hairpin RNA for human circLATS2 (Sh-circLATS2), pcDNA3.0 vector for overexpressing circLATS2 (circLATS2), Sh-E2F7, and negative control. miR-520a-3p mimics and negative controls were synthesized by RiboBio (Guangzhou, China). The 100 nmol vector was transfected into cells using Lipofectamine 2000 (Invitrogen, UAS). The protein expression was detected after the cells were cultured for 3-4 days.

### 2.3. Specimens

Human liver cancer were collected from 112 HCC patients before or after resection in the Suzhou Ninth People's Hospital. All procedures were approved by the Ethical Committee of the Suzhou Ninth People's Hospital. Written informed content was obtained from every participant prior to study.

### 2.4. Quantitative Real-Time Polymerase Chain Reaction (qRT-PCR)

After total RNA was extracted, cDNA was synthesized by reverse transcription and amplified by SYBR Premix Ex TaqII (Takara Biotechnology Co., Ltd.). GAPDH as internal reference. The detection methods of miR-520a-3p were as follows: total RNA was extracted, and cDNA was synthesized by reverse transcription. U6 was used as the internal reference, and PCR was amplified by TaqMan probe method. The 2^−ΔΔCt^ method was adopted to calculate relative expression. The experiment was independently repeated for 3 times, with 3 repeated holes each time.

### 2.5. Fluorescence In Situ Hybridization (FISH)

FISH detection Alexa Fluor 555-labeled probe synthesized by GenePharma was used to detect circRNALATS2. Cells were washed with CSK buffer containing 0.5% TRiton X-100 and 10 mM VRC. After fixed with 4% paraformaldehyde, the cells were incubated with prehybridization buffer and hybridized at 55°C for 2 h. The nuclei were then stained with DAPI. According to the manufacturer's instructions, the probe signal was measured using a FISH kit (RiboBio, Guangzhou, China) and observed under a fluorescence microscope.

### 2.6. Dual-Luciferase Reporter Assay

The sequence containing the binding site fragment of circLATS2 and miR-520a-3p was constructed into the pmirGLO plasmid (Promega, Madison, WI, USA) to obtain the plasmid circLATS2-WT, binding site mutation (Mut, UGUAUUUUAUCCAUAGGAGUUA), and construction of mutant plasmid circLATS2-MUT. The plasmids were transfected into HEK-293T cells with miR-520a-3p mimic, and the luciferase intensity was determined by using dual-Luciferase Reporter Assay Kit (Promega). The experiment was independently repeated for 3 times, with 3 repeated holes each time. The experimental steps of E2F7 and miR-520a-3p were the same as above.

### 2.7. RNA Pull-Down

The cells were lysed and sonographed. Biotinylated miR-520a-3p probes (Genepharm, Shanghai, China) and Streptavidin Agarose beads (Thermo Scientific) were incubated at 25°C for 2 hours to generate probe-coated magnetic beads. Lysis cells with miR-520a-3p probe were incubated overnight at 4°C. Then, the RNA complexes bound to the magnetic beads were eluted with eluent, and the RNA bound to the magnetic beads was captured and extracted with Trizol. The content was determined by RT-PCR using RNeasy Mini Kit (QIAGEN). The experiment was independently repeated for 3 times, with 3 repeated holes each time.

### 2.8. Clone Formation Assay

The cells were seeded in 6-well plates, and the medium was changed every other day. Culture was stopped when visible cells fell behind in the dish. The cells were fixed with precooled 4% paraformaldehyde for 15 minutes and stained with crystal violet for 30 minutes. The number of cells formed by cloning was counted under the microscope.

### 2.9. Transwell Assay

The liquefied Metrigel was placed in the upper chamber of the 24-well Transwell inserts and left to solidified in an incubator. The cells were inoculated with (invasion) or without Matrigel-covered inserts, and placed in 24-well plates. Medium containing 20%FBS was added to the lower chamber, and medium containing 10%FBS was added to the upper chamber. After 24 hours, the Transwell inserts was removed and fixed with 4% paraformaldehyde. Then, crystal violet staining was used, and residual cells in the upper ventricle were wiped with cotton swabs. Microscopically, the invasions were counted. The experiment was independently repeated for 3 times, with 3 repeated holes each time.

### 2.10. Cell Counting Kit-8 (CCK-8) Assay

The cells were inoculated in 96-well plates and cultured in an incubator for 4 hours. After that, CCK8 reagent was added and cultured for another 3 hours. The absorbance was measured at 450 nm. The experiment was independently repeated for 3 times, with 3 repeated holes each time.

### 2.11. Western Blot Assay

Cellular proteins were extracted with RIPA lysis buffer (Sigma Aldrich), separated using SDS-PAGE (Beyotime), and transferred to polyvinylidene fluoride membranes (Beyotime). Membranes were then blocked in 5% skim milk and treated with anti-E2F7 (1 : 1000, ab56022; Abcam, UK), VEGFR2 (1 : 300, bs-10412R; Bioss, China), p-VEGFR2 (1 : 1000, ab5473; Abcam, UK), GAPDH (1 : 3000, bs-2188R; Bioss, China), and mouse anti-rabbit secondary antibody (1 : 5000, bs-0295M-HRP; Bioss, China). Western blots were visualized using the ECL system (Beyotime).

### 2.12. Immunohistochemistry (IHC) Assay

Tumor paraffin sections were successively dewaxed and hydrated, antigen repair, serum blocking, CD34 antibody (1 : 2500, ab81289; Abcam, UK) staining-secondary antibody (BS-0295M-HRP; (Bioss, China) incubation-DAB coloration-gradient alcohol dehydration-neutral gum sealing sheet. The protein expression was observed under a microscope.

### 2.13. Murine Xenograft Model

BALB/c nude mice were purchased from Beijing Vital River Laboratory Animal Technology Co., Ltd. (Beijing, China). The mice were treated with sh-NC, sh-circLATS2, vector-NC, or sh-circLATS2 transfected cells (*n* = 3). Tumor volume was measured daily 7 days after cell implantation. The mice were euthanized after 28 days, and the tumor weight was weighed. This study was approved by the Ethics Committee of Suzhou Ninth People's Hospital.

### 2.14. Database

CircInteractome (https://circinteractome.nia.nih.gov/) was used to predict circRNA potential of miRNAs and their potential binding sites, using the TargetScan (http://www.targetscan.org/vert_71/) to predict the miRNA potential target genes and its binding site.

### 2.15. Statistical Analysis

Statistical analysis was implemented using GraphPad Prism 7 (GraphPad Inc., La Jolla, CA, USA). The chi-square test was used for the analysis of the enumeration data, and the *t* test was used for the measurement data. One-way ANOVA after post hoc test was used for comparison between multiple groups. The comparison of means was done via Student's *t*-test or one-way analysis of variance. *P* < 0.05 was thought to be significant.

## 3. Results

**Figure 1:**
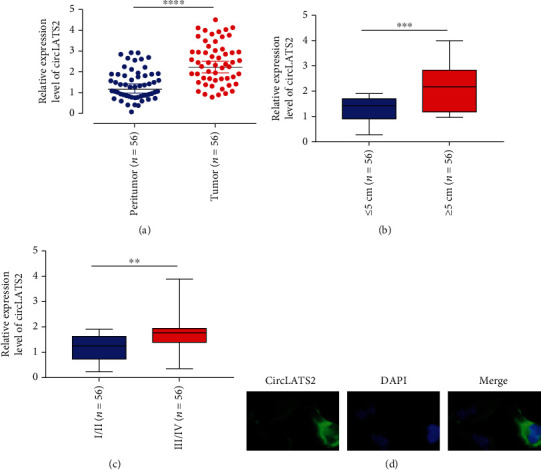
The expression of circLATS2 is upregulated in HCC. CircLATS2 relative expression levels in HCC tissues (a), different HCC tissue sizes (b), different stages of HCC (c). (d) FISH detected the intracellular expression and localization of circLATS2. HCC: hepatocellular carcinoma; FISH: fluorescence in situ hybridization.

The expression of circLATS2 is upregulated in HCC. We first detected the expression of circLATS2 in HCC tissues and adjacent normal tissues. The results showed that tumor tissue had significantly higher circLATS2 expression than normal tissue (Figure 1(a)). Besides, the groups were compared according to tumor tissue size. It was found that circLATS2 was significantly overexpressed when the tumor tissue was ≥5 cm (Figure 1(b)). We then compared the expression of different tumor stages and found that the level of expression was higher in tumor tissues of stage III/IV (Figure 1(c)). Finally, we detected the expression of circLATS2 in the cytoplasm of tumor cells by FISH assay (Figure 1(d)).

### 3.1. CircLATS2 Served as the Sponge for miR-520a-3p

**Figure 2:**
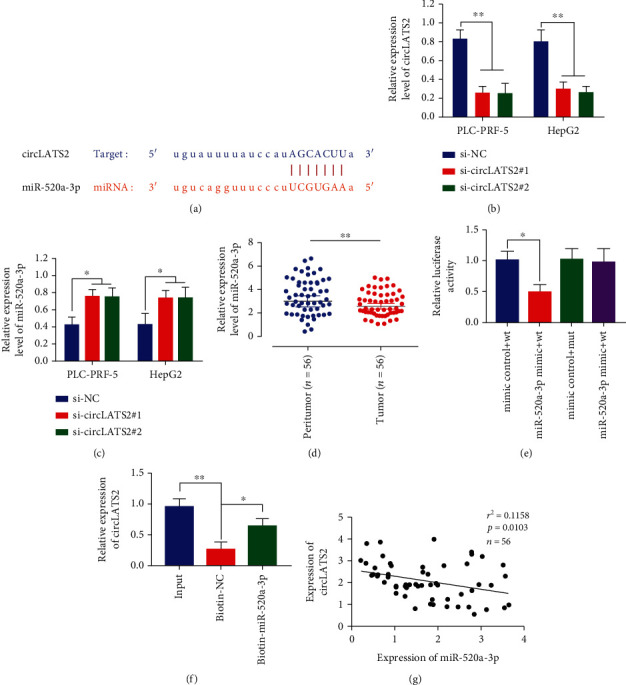
CircLATS2 is a ceRNA of miR-520a-3p in HCC. (a) Binding sites of circLATS2 and miR-520a-3p. (b) Expression level of circLATS2 in PLC-PRF-5 and HepG2 cells. (c) Effect of circLATS2 on expression level of miR-520a-3p. (d) miR-520a-3p expression levels in paracancer and HCC tissues. The binding of CircLATS2 to miR-520a-3p was determined by dual luciferase reporter gene assay (e) and RNA pull-down (f). (g) Correlation between circLATS2 and miR-520a-3p. HCC: hepatocellular carcinoma.

We screened the miRNA used by circLATS2 and found miR-520a-3p. The binding sites of circLATS2 and miR-520a-3p are shown in Figure 2(a). We detected the expression of circLATS2 in PLC-PRF-5 and HepG2 HCC cell lines and found that the expression of Si-circLATS2 was significantly reduced compared with the control group (Figure 2(b)). We detected the expression of miR-520a-3p by circLATS2, and the results showed that the expression of miR-520a-3p was significantly increased after circLATS2 was knocked out (Figure 2(c)). Then, miR-520a-3p was detected in cancer tissues and paracancer tissues, and it was found that miR-520a-3p was highly expressed in HCC tissues compared with paracancer tissues (Figure 2(d)). What is more, the relationship between circLATS2 and miR-520a-3p was verified by dual luciferase reporter gene assay and RNA pull down, and the two were found to interact (Figures 2(e) and 2(f)). Subsequently, we tested the correlation between the circLATS2 and miR-520a-3p, and Figure 2(g) showed that the expressions were negatively correlated. Collectively, circLATS2 sponged miR-520a-3p to its expression.

### 3.2. CircLATS2 Acts on miR-520a-3p and Affects the Expression of E2F7

**Figure 3:**
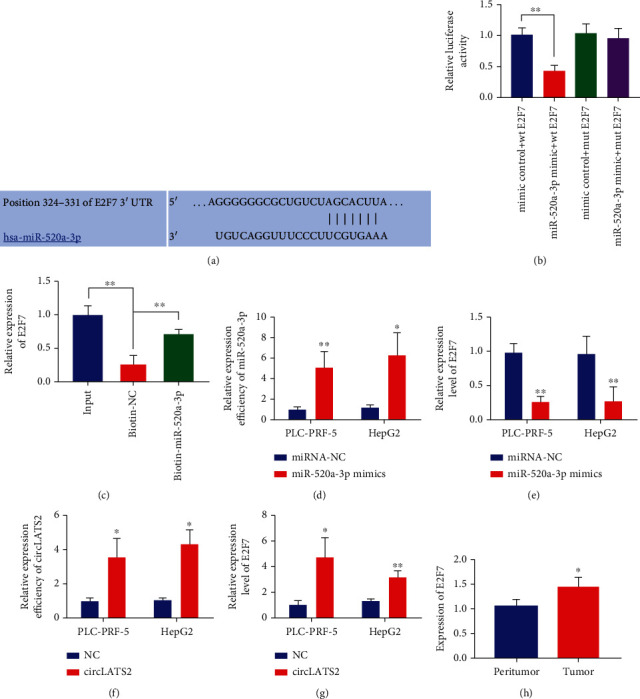
miR-520a-3p targets E2F7. (a) Binding sites of miR-520a-3p and E2F7. The binding of miR-520a-3p to E2F7 was determined by dual luciferase reporter gene assay (b) and RNA pull-down (c). (d) qRT-PCR was used to detect the transfection efficiency of miR-520a-3p mimics in PLC-PRF-5 and HepG2 cells. (e) The effect of miR-520a-3p on E2F7 expression was detected by qRT-PCR. (f) qRT-PCR was used to detect the transfection efficiency of circLATS2. (g). The effect of circLATS2 on E2F7 expression was detected by qRT-PCR. (h) qRT-PCR was used to detect the expression level of E2F7 in paracancer and HCC tissues. HCC: hepatocellular carcinoma; qRT-PCR: quantitative real-time polymerase chain reaction.

We predicted the downstream target of miR-520a-3p, discovered E2F7, and predicted the binding sequence of the two (Figure 3(a)). Reporter gene assay and RNA pull down assay showed that they had an interaction (Figures 3(b) and 3(c)). Then, we overexpressed the content of HCC cell line miR-520a-3p and verified the transfection efficiency (Figure 3(d)). In addition, we verified the effect of cell line miR-520a-3p on E2F7 expression. The results showed that when miR-520a-3p was overexpressed, the expression of E2F7 was significantly reduced (Figure 3(e)). In order to verify the influence of circLATS2 on E2F7 expression, we overexpressed circLATS2 and verified its transfection efficiency and then detected the expression of E2F7. The results suggested that E2F7 expression was decreased when circLATS2 was overexpressed (Figure 3(g)). Moreover, we detected the expression of E2F7 in HCC and adjacent tissues and found that it was significantly overexpressed in patients with HCC (Figure 3(h)).

### 3.3. Low Expression of circLATS2 Inhibits the Malignancy of HCC Cells

**Figure 4:**
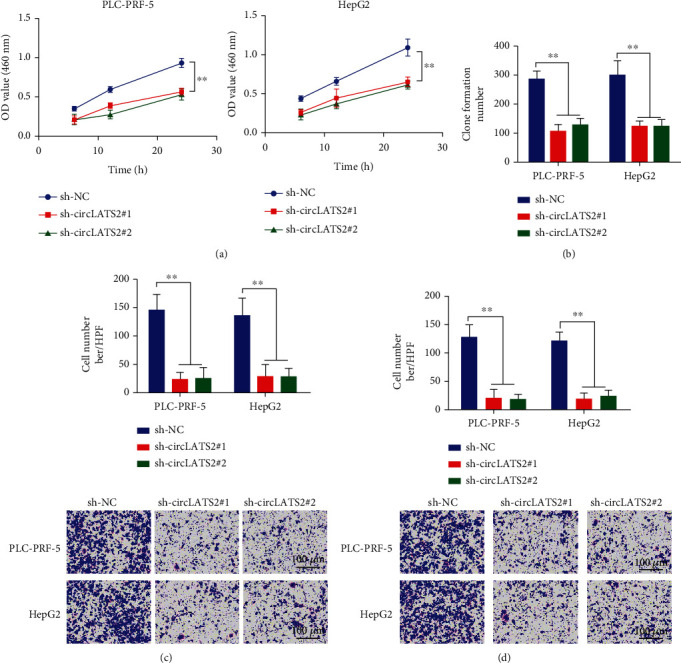
Knockdown circLATS2 inhibited the malignant behavior of HCC cell in vitro experiments. (a) CCK-8 assay was used to detect the effect of circLATS2 on cell proliferation. (b) The effect of circLATS2 on cell clone formation ability was detected. Transwell assay was used to detect the effects of circLATS2 on cell migration (c) and invasion (d). HCC: hepatocellular carcinoma; CCK-8: Cell Counting Kit-8.

We verified the effects of circLATS2 on proliferation, clonal formation, migration, and invasion of HCC cells. The results showed that the proliferation, clonal formation, invasion, and migration of tumor cells were significantly reduced when circLATS2 was knocked out (Figure 4).

### 3.4. CircLATS2 Knockdown Alleviated Tumor Growth In Vivo

**Figure 5:**
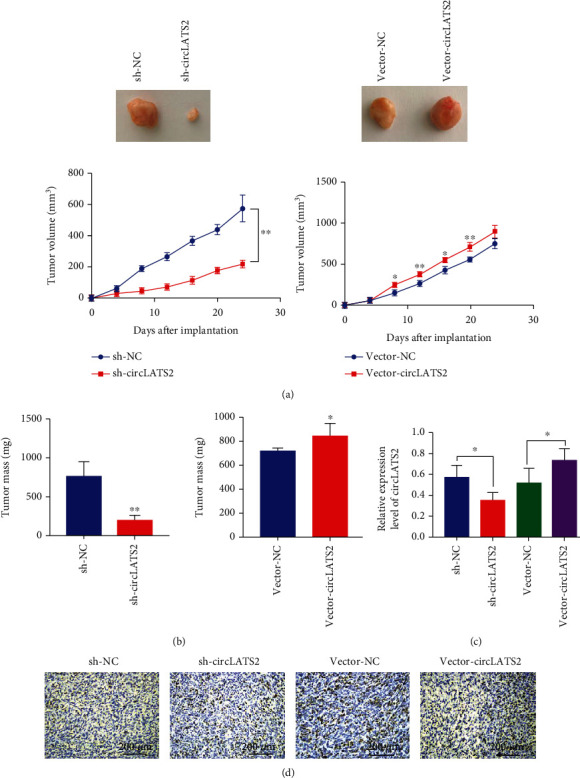
Knockdown circLATS2 inhibits the proliferation of HCC cells, while overexpression of circLATS2 promotes the proliferation of HCC cells in vivo. Tumor proliferation curve and tumor weight in nude mice (a, b). (c) The expression level of circLATS2 in tumor tissue. (d) The expression of CD34 was detected by immunohistochemistry. HCC: hepatocellular carcinoma.

We established a nude mouse HCC transplanted tumor model and determined the effect of circLATS2 on tumor cells through knockdown and overexpression. The results showed that when circLATS2 was knocked down, tumor growth slowed down and tumor weight decreased (Figures 5(a) and 5(b)). Additionally, we detected the expression of circLATS2 in tumor tissues, and the results showed that the overexpression group was significantly higher than the knockout group (Figure 5(c)). Of note, immunohistochemistry showed that the expression of CD34 was significantly higher in the overexpression group than in the knockout group (Figure 5(d)).

### 3.5. Overexpression of miR-520a-3p and E2F7 Knockdown Reversed the Promoting Effect of circLATS2 on HCC

**Figure 6:**
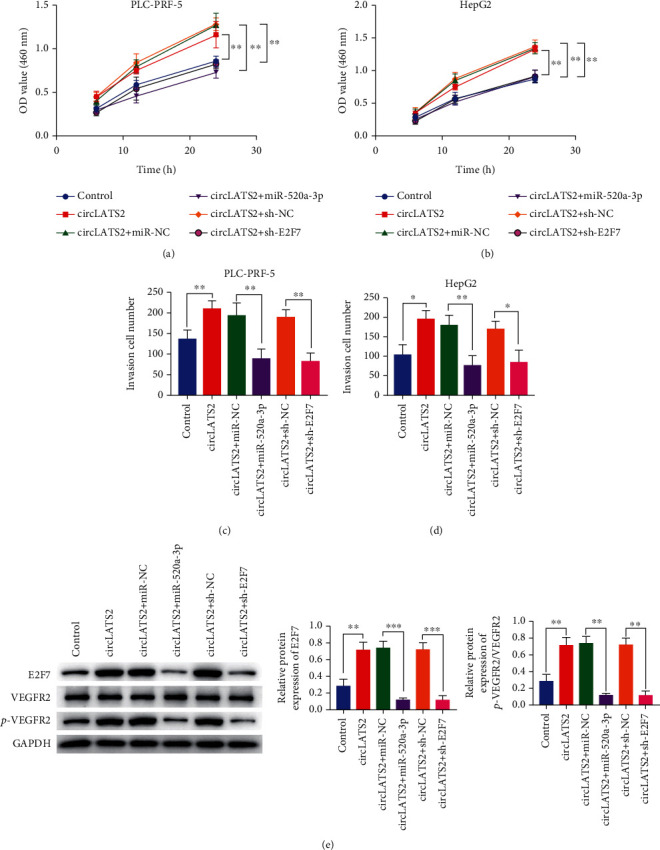
Overexpression of miR-520a-3p and E2F7 knockdown reversed the promoting effect of circLATS2 on HCC. The proliferation of PLC-PRF-5 (a) and HepG2 (b) cells was detected by CCK-8 assay. The invasiveness of PLC-PRF-5 (c) and HepG2 (d) cells was detected by Transwell assay. (e) Western blot was used to detect the expression levels of E2F7, VEGFR2, and P-VEGFR2. HCC: hepatocellular carcinoma; CCK-8: Cell Counting Kit-8.

We constructed cell lines that overexpressed circLATS2 and miR-520a-3p, as well as overexpressed circLATS2 and knockdown E2F7, and verified their effects on the proliferation and invasion of tumor cells through CCK8 and Transwell assay. The results showed that when circLATS2 and miR-520a-3p were overexpressed at the same time, the proliferation and invasion abilities of cells were significantly reduced compared with overexpressed circLATS2. The same results were also found in the overexpressed circLATS2 and knockdown E2F7 groups (Figures 6(a)–6(d)). We then examined the protein levels of E2F7, VEGFR2, and P-VEGFR2 in cells, suggesting that the phosphorylation of VEGFR2 is reduced when circLATS2 and Mir-520a-3p are overexpressed simultaneously. The same result was obtained when circLATS2 was overexpressed and E2F7 was knocked down (Figure 6(e)).

## 4. Discussion

circRNAs are mainly regarded as microRNA sponges, protecting their target genes from cleavage by certain miRNAs [19]. Additionally, circRNA-miRNA networks are closely related to the occurrence, development and prognosis of cancer [20]. In recent years, there are more and more studies on circRNA in tumors, including bladder cancer, gastric cancer, and cervical cancer. Circ-BPTF promotes bladder cancer progression and recurrence through miR-21-5p/RAB27A axis, while Circ_0008035 promotes gastric cancer cell proliferation and inhibits apoptosis through miR-599/EIF4A1 axis [21, 22]. In cervical cancer, circ-E2F3 inhibits miR-296-5p increasing STAT3 nuclear translocation and upregulates expression of cyclin D1 [23]. Similar studies have been conducted in hepatocellular carcinoma. Yang et al. found that circ-CSPP1 inhibited the progression of liver cancer by upregulating miR-493-5p and downregulating HMGB1 through in vitro experiments [24]. Luo et al. found that hsa_circ_0013290 overexpressed in the cytoplasm of hepatocellular carcinoma cell lines can promote the migration and invasion of cells and inhibit their apoptosis [25].

In this study, a novel circRNA-CircLATS2 was explored, and its role in liver cancer was validated. The results of this study showed that circLATS2 was highly expressed in HCC tissues and increased with the increase of tumor volume and tumor stage. At the same time, we found that miR-520a-3p was significantly lower expressed in HCC tissues, and when circLATS2 was knocked out, the expression of miR-520a-3p was significantly increased. CircLATS2 served as the sponge for miR-520a-3p. We then found that circLATS2 targets E2F7 through miR-520a-3p to affect HCC cells. We demonstrated at the cellular level that knockdown circLATS2 inhibits the proliferation, clone formation ability, migration, and invasion of HCC cells. Meanwhile, the overexpression of miR-520a-3p and the knockdown of E2F7 reversed the promoting effect of circLATS2 on HCC. In vivo, tumor growth and weight were inhibited when circLATS2 was knocked out. It was reversed when circLATS2 was overexpressed. It was demonstrated that circLATS2 served as an oncogenic drive in HCC via miR-520a-3p/E2F7 pathway.

When circLATS2 was knocked out, the proliferation, clone formation, migration, and invasion ability of HCC cells were inhibited. After that, we constructed a nude mouse transplanted tumor model to determine the effect of circLATS2 on hepatocellular carcinoma in vivo and found that circLATS2 knockdown inhibited tumorigenesis, while overexpression was opposite. All these findings indicated circLATS2 enhanced malignant phenotypes of hepatocellular carcinoma cells. Subsequently, we found that miR-520a-3p was erased by circLATS2 to influence the malignant behavior of HCC cells. miR-520a-3p, as a tumor suppressor gene, can inhibit the proliferation of ovarian cancer, cervical cancer, and gastric cancer [26–28]. In this study, miR-520a-3p was sponged by circLATS2 as a tumor suppressor gene, similar to the results of Wang et al. [29]. Our results showed that overexpressed miR-520a-3p could reverse the inhibitory effect of circLATS2 on HCC, including cell proliferation, migration, and invasion.

E2F is an important transcription factor for tumor growth. E2F7 is an atypical E2F factor that can promote the proliferation and migration of liver cancer and glioblastoma [30, 31]. Moreno et al. constructed transgenic mice with doxycycline-controlled transcriptional activation of E2F7. The study had found that E2F7-dependent transcription is essential for the proliferation of mammalian cells. When combined with the inhibition of E2F-dependent transcription in the S phase and DNA destruction reagent, the synergistic killing of cancer can be achieved [32]. Our results show that E2F7 is highly expressed in tumor tissues of HCC patients. miR-520a-3p affects the malignant behavior of HCC cells by targeting the inhibition of E2F7 expression. Similarly, Teng et al. found that miR-424-5p activated the VEGFR-2 signaling pathway of hepatocellular carcinoma by targeting E2F7 and promoted tumor angiogenesis [33]. This study also clarified the effects of E2F7 on malignant behavior and angiogenesis-related factors of hepatocellular carcinoma. Knockdown of E2F7 reversed the effects of circLATS2 on the proliferation and invasion of HCC and also reduced the phosphorylation level of VEGFR2, activating VEGFR2 signaling pathway. In conclusion, our study suggests that miR-520a-3p can reverse the promoting effect of circLATS2 on HCC by targeting the E2F7-VEGFR2 signaling pathway.

Vascular morphology was not studied in this study, and there are insufficient results to clarify whether circLATS2 has an effect on vascular formation. In addition, the downstream pathways of E2F7/VEGFR2 have not been further elucidated.

Taken together, our study confirmed that circLATS2 promotes the proliferation, migration, and invasion ability of HCC cells through miR-520a-3p/E2F7-VEGFR2 signaling pathway, which may be a potential target for HCC therapeutic.

## Data Availability

The data used to support the findings of this study are available from the corresponding author upon request.
